# Effect of biochemical and biomechanical factors on vascularization of kidney organoid-on-a-chip

**DOI:** 10.1186/s40580-021-00285-4

**Published:** 2021-11-08

**Authors:** Han Na Lee, Yoon Young Choi, Jin Won Kim, Young Seo Lee, Ji Wook Choi, Taewook Kang, Yong Kyun Kim, Bong Guen Chung

**Affiliations:** 1 Department of Biomedical Engineering, Sogang University, Seoul, South Korea; 2 Institute of Integrated Biotechnology, Sogang University, Seoul, South Korea; 3 Cell Death Disease Research Center, College of Medicine, The Catholic University of Korea, Seoul, South Korea; 4 Department of Mechanical Engineering, Sogang University, Seoul, South Korea; 5 Department of Chemical and Biomolecular Engineering, Sogang University, Seoul, South Korea; 6 Department of Internal Medicine, College of Medicine, The Catholic University of Korea, St. Vincent’s Hospital, Suwon, South Korea

**Keywords:** Kidney organoid-on-a-chip, Shear stress, Vascular structure, Podocyte

## Abstract

Kidney organoids derived from the human pluripotent stem cells (hPSCs) recapitulating human kidney are the attractive tool for kidney regeneration, disease modeling, and drug screening. However, the kidney organoids cultured by static conditions have the limited vascular networks and immature nephron-like structures unlike human kidney. Here, we developed a kidney organoid-on-a-chip system providing fluidic flow mimicking shear stress with optimized extracellular matrix (ECM) conditions. We demonstrated that the kidney organoids cultured in our microfluidic system showed more matured podocytes and vascular structures as compared to the static culture condition. Additionally, the kidney organoids cultured in microfluidic systems showed higher sensitivity to nephrotoxic drugs as compared with those cultured in static conditions. We also demonstrated that the physiological flow played an important role in maintaining a number of physiological functions of kidney organoids. Therefore, our kidney organoid-on-a-chip system could provide an organoid culture platform for in vitro vascularization in formation of functional three-dimensional (3D) tissues.

## Introduction

Kidney organoids are emerged in recent years in a pre-clinical, post-approval stage of the pharmaceutical development [1]. The human pluripotent stem cells (hPSCs)-derived human kidney organoid is a novel method of a three-dimensional (3D) culture in vitro model for drug screening applications. Under appropriate induction (e.g., growth factor treatment), the stem cells could differentiate into the specific kidney cell lineages. The stem cell-induced kidney organoids have the similar structure and physiology to a human kidney tissue [2]. It allows the recapitulation of the physical structure and physiological functions of the kidney in vivo [3]. Additionally, the human kidney organoid model can be scale-up for high-throughput drug screening at a low cost without any ethical concern as compared to traditional animal models [4, 5].

Despite the great potential of the human kidney organoids, the kidney organoids are functionally immature and anatomically different from in vivo human tissues at a current stage. The major concerns about the generation of kidney organoids from hPSCs are the lack of the vascularization which still limits the growth and maturation of the organoids [6]. Organoids in a 3D culture solely rely on the passive diffusion to receive the nutrients and oxygen as well as remove the waste products. For in vivo situations, most cells cannot survive when located more than 200 μm away from a blood vessel due to the limited diffusion of the oxygen and nutrients [7]. Thus, the vascularization in vitro, *which* can prevent the cell death, enables the construction of the large engineered tissues, such as the heart, liver, and kidney. Furthermore, the endothelium of the vasculature allows for the paracrine signaling and basement membrane interactions with other cell types, which could potentially improve the organoid maturation [8]. The embryogenesis was tightly coupled with vascular development and the functional vasculature capable of delivering adequate blood was also required for later stages of organogenesis [9]. Therefore, the vascularization of the organoids is emerging as a promising strategy to address the problem of limited nutrient supply and life span imposed by the traditional organoid models.

The blood vessel formation of the organoids is a complex process which requires the biochemical and biomechanical factors, such as growth factors, extracellular matrix (ECM) components, and in vivo-related physiological stimulations. The composition and properties of ECM, a complex network of large molecules, are highly variable, affecting the tissue-specific differentiation of the stem cells. Furthermore, ECM contributes the maintaining of the blood vessel structure and elasticity, regulating the proliferation and differentiation of endothelial cells (ECs) and transporting the angiogenic growth factors [10]. Additionally, the EC differentiation and the formation of blood vessels is generally controlled by several protein factors including fibroblast growth factor 2 (FGF-2) [11, 12], bone morphogenic protein-4 (BMP-4) [13], and vascular endothelial growth factor (VEGF) [14]. Among these factors, a VEGF is a fundamental regulator in the differentiation, migration, and cell–cell interactions of ECs including stimulation of sprouting of angiogenesis and activation of tip cells [15]. Furthermore, a VEGF produces the chemical signals that can regulate the directional migration of newly forming vessel capillaries [16].

The blood vessel formation is subjected to the traction stresses generated by peri-vasculature cells and viscoelastic properties from neighboring ECMs [7]. In particular, the ECs are also subjected to the shear stress induced by various flow types (e.g., pulsatile or unidirectional blood flow, transmural flow, and interstitial flow) [17]. The shear stress generated by the blood flow on the luminal surface of ECs is a mechanical factor that induces intra cellular biochemical pathways, resulting in the change of the gene expression as well as the modulation of the structure and function of blood vessels [18]. Although the biomechanical factors are as important as biochemical factors in the formation of the blood vessels and mature organoids, they have not been fully studied yet due to the complexity of engineering culture systems to investigate or implement individual mechanical factors. Thus, the lack of the biomechanical manipulation has been recognized as a critical limitation to the further development of the matured organoids. To address this problem, a number of studies have recently established the microfluidic culture system that can mimic in vivo-like microenvironments to generate the matured human organoids [19]. Lee et al. has developed a stomach-on-a-chip model using the peristaltic pump that could mimic the luminal flow and rhythmic contraction of the stomach in vivo [20]. Additionally, the millimeter-scale microfluidic system has been developed to examine the effect of the flow on vascularization and maturation of kidney organoids [21]. These biomechanical culture systems could generate physiological biomechanical factors as well as promote structural and functional maturation of developing organoids. Moreover, the shear stress induced the prominent vascularization and maturation of the kidney organoids, showing that the fluidic flow-generated microenvironments could contribute to the structural and functional development of the human kidney organoids in vitro [21]. In our study, we posit that the vascularization and maturation can be promoted in kidney organoids in vitro when it is subjected to biomechanical factors. To prove this hypothesis, we developed a kidney organoid-on-a-chip system to demonstrate the effect of ECM and fluidic shear stress on in vitro development of hPSCs-derived human kidney organoids.

## Materials and methods

### Fabrication of the kidney organoid-on-a-chip

The kidney organoid-on-a-chip was fabricated using a mold produced by a 3D printer (DP120, WOW3D, Korea) as previously described [22, 23]. Briefly, a 3D printed mold was designed by AutoCAD software and the modeling data were transferred to the 3D printing software. Afterwards, an ultraviolet (UV)-curable resin was cured for the mold fabrication. The resulting mold was washed by isopropyl alcohol (IPA, Sigma Aldrich, USA) and was subsequently dried by air blowing to remove the residual uncured resin. Poly(dimethylsiloxane) (PDMS, Sylgard184, Dow Corning, USA) was mixed in a ratio of 10:1 (monomer: curing agent), degassed, poured onto the fabricated mold, and baked at 80 ℃ for 2 h. The cured PDMS chip was carefully detached from the mold and was subsequently bonded onto the slide glass (Marienfeld, Germany) using O_2_ plasma treatment (Femto Scientific, Korea). The top side of the chip was then covered by a piece of PDMS mold with the inlet and outlet.

### Computational fluid dynamics (CFD) simulation

Before the experiments, the performance of the kidney organoid-on-a-chip was simulated using CFD model to investigate the fluid velocity distribution and shear stress acting on the organoid surface. The microchannel modeling was performed with Autodesk Inventor (Autodesk Inc. USA) 3D CAD software and was subsequently imported to COMSOL Multiphysics 5.5 (Comsol Inc., USA) software to conduct the fluidic flow modeling. The fluid was assumed to be water and the microchannel wall was set to no slip condition. The shape of kidney organoid was modeled as an elliptical sphere with a height of 400 μm, a major axis of 500 μm, and a minor axis of 400 μm. In the microchannel, 10 cylindrical microwells were evenly located and the kidney organoids were occupied in an each well. To determine the effect of microwell dimensions on the fluidic flow velocity profile and the wall shear stress on the kidney organoid surface, the diameter and height of the microwells were defined in the range of 600 μm to 2.1 mm and 100 μm to 2 mm, respectively. We calculated the fluidic flow velocity profile by assuming stationary (steady-state), single-phased laminar flow (SPF), with the inflow rates set to 1, 5, 10, 20, and 30 µL/min, respectively. Within the microchannel, the flow velocity $u$ is dominated by the homogeneous, incompressible Naiver–Stokes equation and continuity equation:

$$\rho \left(u\cdot \nabla \right)u=\nabla \cdot \left[-p{\rm I}+\tau \right]+F$$

$$\rho \nabla \cdot u=0$$

where $\rho$ is the fluid density, $u$ is the velocity field, $\nabla$ is the divergence, $p$ is the pressure, $\tau$ is the Stoke’s stress ($\tau =\mu (\nabla u+\nabla {u}^{T})$), $\mu$ is the dynamic viscosity, and $F$ is the volume force. Afterward, the shear stress acting on the kidney organoid surface is calculated with the equation ${{\uptau }}_{\text{w}}=\mu \cdot \dot{\gamma }$, where $\mu$ is the dynamic viscosity and $\dot{\gamma }$ is the shear rate at the boundary of kidney organoid surfaces.

### HPSC culture and kidney organoid differentiation

The kidney organoid differentiation was performed as previously described [24]. In brief, hPSCs were plated at a density of 5000 cells/well on a 24-well plate in mTeSR1 medium (Stem Cell Technologies, Vancouver, Canada) plus 10 µM Y27632 (LC Laboratories, Canada) on glass plates (LabTek) coated with 3% Matrigel (Thermo Fisher Scientific, MA, USA). The medium was exchanged for 1.5% Matrigel in mTeSR1, mTeSR1, RPMI (Thermo Fisher Scientific, MA, USA) plus 12 µM CHIR99021 (Tocris, UK), or RPMI plus B27 supplement (Thermo Fisher Scientific, MA, USA). The cells were fed every 2–3 day to promote kidney organoid differentiation. Kidney organoids were seeded into kidney organoid-on-a-chip on day 16.

### Preparation of kidney organoid-on-a-chip

Kidney organoid-on-a-chip was sterilized by autoclaving at 120 °C for 30 min and were subsequently dried in 80 °C oven. The kidney organoid-on-a-chip was coated with 1.5% Matrigel containing 100 ng/mL VEGF for overnight to improve the organoid adhesion. After coating, the kidney organoid-on-a-chip was rinsed three times with Dulbecco’s phosphate-buffered saline (DPBS) before seeding kidney organoids.

### Immunostaining

hPSCs-derived kidney organoids cultured within kidney organoid-on-a-chip were analyzed immunocytochemically to confirm the spatial distribution of kidney-related cells. After culturing for 16 days within petri dishes, the cells were retrieved and were subsequently cultured with kidney differentiation medium under a fluidic flow condition in a kidney organoid-on-a-chip for an additional 6 days. The cells cultured in a kidney organoid-on-a-chip were fixed for 30 min with 8% paraformaldehyde (Electron Microscopy Sciences, Hatfield, PA) at 4 °C. The fixed organoids were blocked in 5% donkey serum (Millipore, USA) plus 0.3% Triton-X‐100/PBS, and incubated overnight in 3% bovine serum albumin (BSA, Sigma Aldrich, MO St. Louis. USA) plus PBS with primary antibodies. Primary antibodies against the following proteins were used to characterize various kidney-related cell types: podocytes (anti-NPHS1, R&D AF4269, 1:500), proximal tubular cells (anti-LTL, Vector Labs FL‐1321, 1:500), and ECs (anti-PECAM1, Abcam ab9498, 1:200). After incubating overnight, kidney organoids were washed with PBS twice. Secondary antibodies (1:500 dilutions, Invitrogen, CA, USA) were applied at room temperature for overnight. Each kidney organoid was washed with PBS and fluorescent images were acquired by using a confocal microscope (Olympus, Japan) after counterstaining with 4,6-diamidino-2-phenylindole dihydrochloride (DAPI, Invitrogen, CA).

### Nephrotoxicity analysis

Tacrolimus (Sigma Aldrich, MO St. Louis. USA) was made up as a 1.8 mM stock solution in dimethyl sulfoxide (DMSO). The reagent was diluted in a culture medium to make an appropriate final concentration. Briefly, the kidney organoids cultured for 6 days in a kidney organoid-on-a-chip were treated 0, 30, and 60 µM tacrolimus concentration for 24 h. After incubation, the kidney organoids transferred into a 96-well plate. The nephrotoxicity was assessed by using a cell counting kit-8 (CCK-8. Dojindo, JAPAN) according to the manufacturer’s procedure. Finally, the OD at 450 nm with a reference wavelength of 690 nm for each sample was measured using a microplate reader (EL800, Bio-Tek Instruments, Winooski, VT, USA).

### Live/dead assay

The viability of tacrolimus-treated kidney organoids within a kidney organoid-on-a-chip was analyzed by using a live/dead assay after 6 days. (Thermo Fisher Scientific, MA, USA). Briefly, 5 mL of DPBS containing 2 µL of calcein AM solution and 10 µL of ethidium homodimer-1 solution was added to kidney organoid-on-a-chip and was then incubated at 37 °C in a 5% CO_2_ incubator for 40 min. The stained organoids were analyzed by using an inverted fluorescence microscope (Olympus, Japan).

### Statistical analysis

The data are expressed as mean ± standard deviation for three times independent experiments. Statistical analysis of the data was performed using the one-way ANOVA or Student’s t-test. Prism 8 (GraphPad, San Diego, CA) was used for all statistical analyses.

## Result and discussion

### Fabrication and modeling of a kidney organoid-on-a-chip

**Fig. 1:**
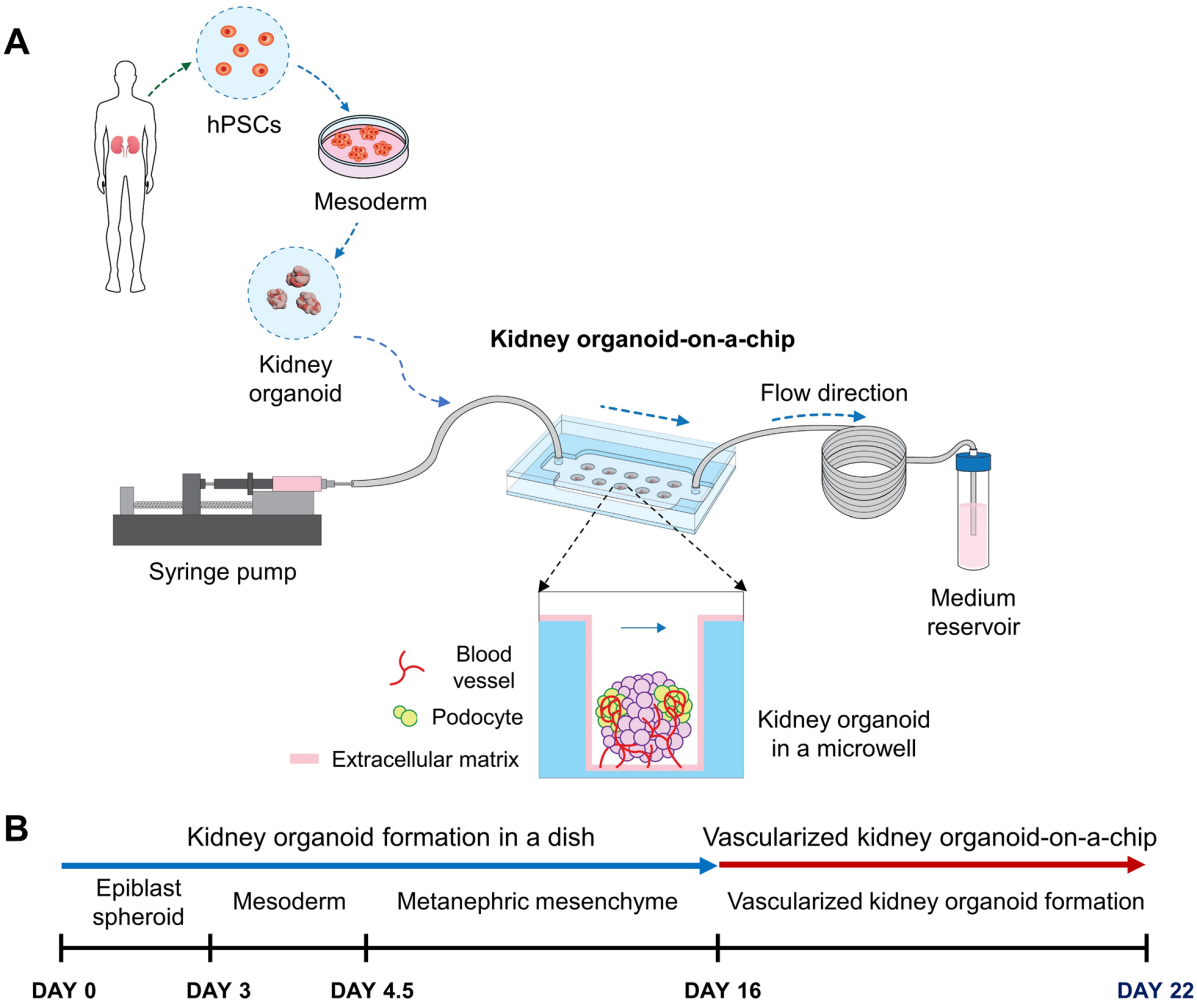
Experimental setup of a kidney organoid-on-a-chip. **A** Induced kidney organoids originated from hPSCs were placed on an optimized ECM and were also subjected to controlled shear stress. **B** Differentiation days and culture conditions to form vascularized kidney organoid-on-a-chip

**Fig. 2:**
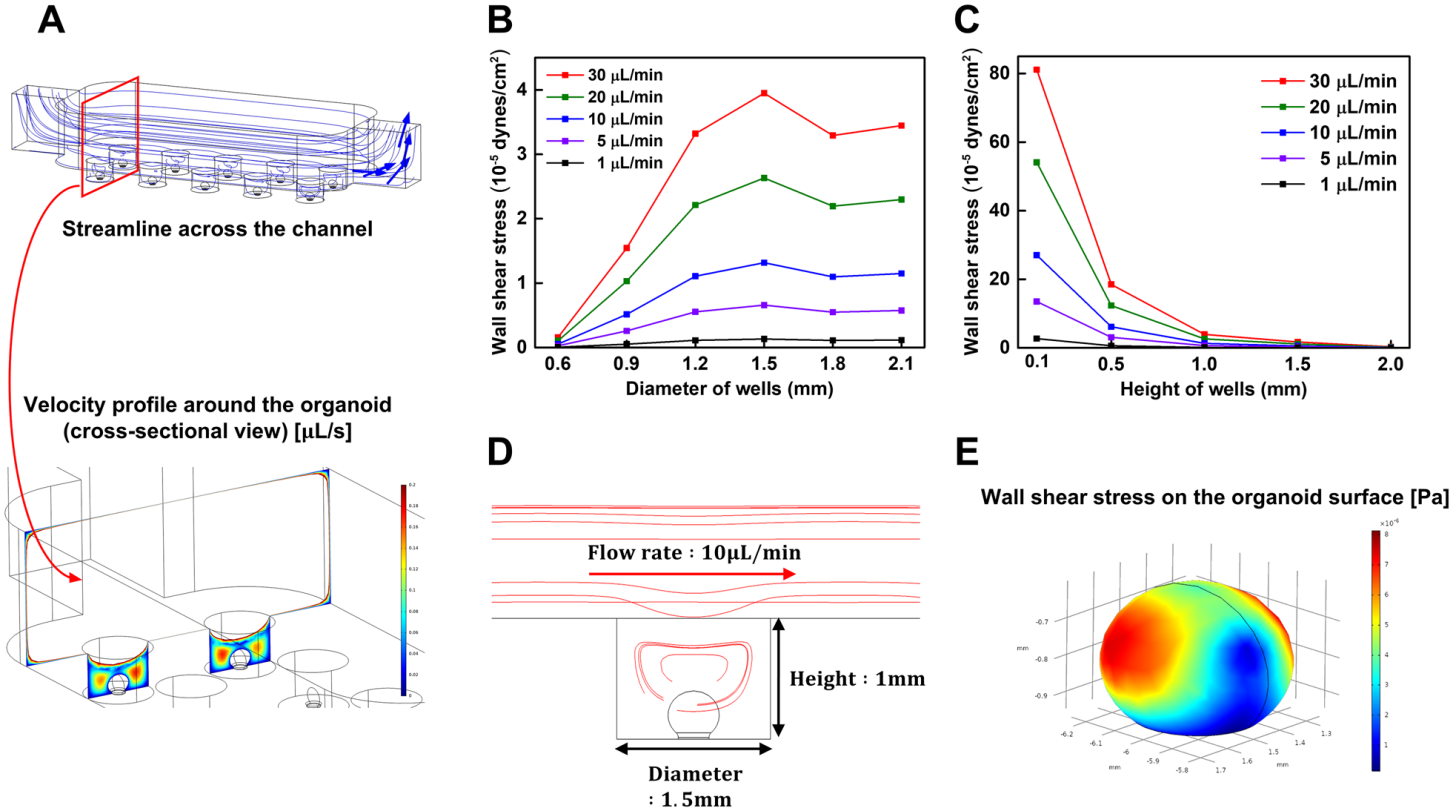
Simulation of kidney organoid-on-a-chip under various fluidic flows and geometric conditions. **A** Simulated streamline profile across the microchannel and cross-sectional velocity profiles around the kidney organoid (bottom). **B** Shear stress acting on the kidney organoid surface with respect to the microwell diameter. **C** Shear stress acting on the kidney organoid surface with respect to the microwell height. **D** Simulated cross-sectional streamline across the microchannel (1 mm well height, 1.5 mm well diameter). **E** Wall shear stress acting on the kidney organoid surface

We fabricated a kidney organoid-on-a-chip system with cylindrical microwells to culture kidney organoids using a 3D printer (Fig. 1A). The dimensions of the cylindrical microwells to culture the kidney organoids were 1 mm height and 1.5 mm diameter, allowing cultivation of 300–500 μm size of kidney organoids per microwell. The design of the kidney organoid-on-a-chip with cylindrical microwells could facilitate the culture of the uniform-sized kidney organoids in compartmentalized microwells. We generated the maturation of hPSCs-derived kidney organoids in the dish and microfluidic systems (Fig. 1B). Prior to the experiment, the computational simulations of the laminar flow inside the microchannel of the kidney organoid-on-a-chip were conducted to predict the wall shear stress acting on the kidney organoid surface using COMSOL software (Fig. 2). Assuming an isotropic Newtonian flow in the microchannel, the shear stress in the fluidic flow was calculated by the following equation [25].

$$\tau \left(\overrightarrow{u}\right)=\mu \nabla \overrightarrow{u}$$

where *µ* is the dynamic viscosity of the fluid and $\nabla \overrightarrow{u}$ is the gradient of velocity vector of the flow which is called as a wall shear rate in a boundary condition. After obtaining the fluidic flow velocity profile in the microchannel by simulation, we calculated the wall shear stress acting on the kidney organoid surface. The dimensions of the kidney organoid modeling were used from the previous study [26]. For higher accuracy of simulation results as compared to two-dimensional (2D) structure, the kidney organoid was designed in 3D structure with the shape of an elliptical sphere [27]. As the fluid flowed through the microchannel, some of streamline passed through the microwell region, expecting to cause higher shear stress on the kidney organoid surface (Fig. 2A). To determine the optimal dimension of the microwells inside the kidney organoid-on-a-chip, we simulated with various heights and diameters of the microwells. From the analysis of the wall shear stress acting on the organoid surface in various flow rates (Fig. 2B), we observed that the maximum wall shear stress was occurred at the 1.5 mm microwell diameter at all flow rates. It is probably due to the geometric characteristics, which eddy occurs inside the microwells and generates higher wall shear stress acting on the kidney organoid surface (Fig. 2D, E) [26]. For simulation to optimize the microwell height, we observed that the wall shear stress was decreased with increasing the microwell height (Fig. 2C). The flow rate and the magnitude of the wall shear stress have a proportional relationship, since all flow rates have the same streamline on simulation.

### Vascularization of kidney organoids in a static condition

**Fig. 3:**
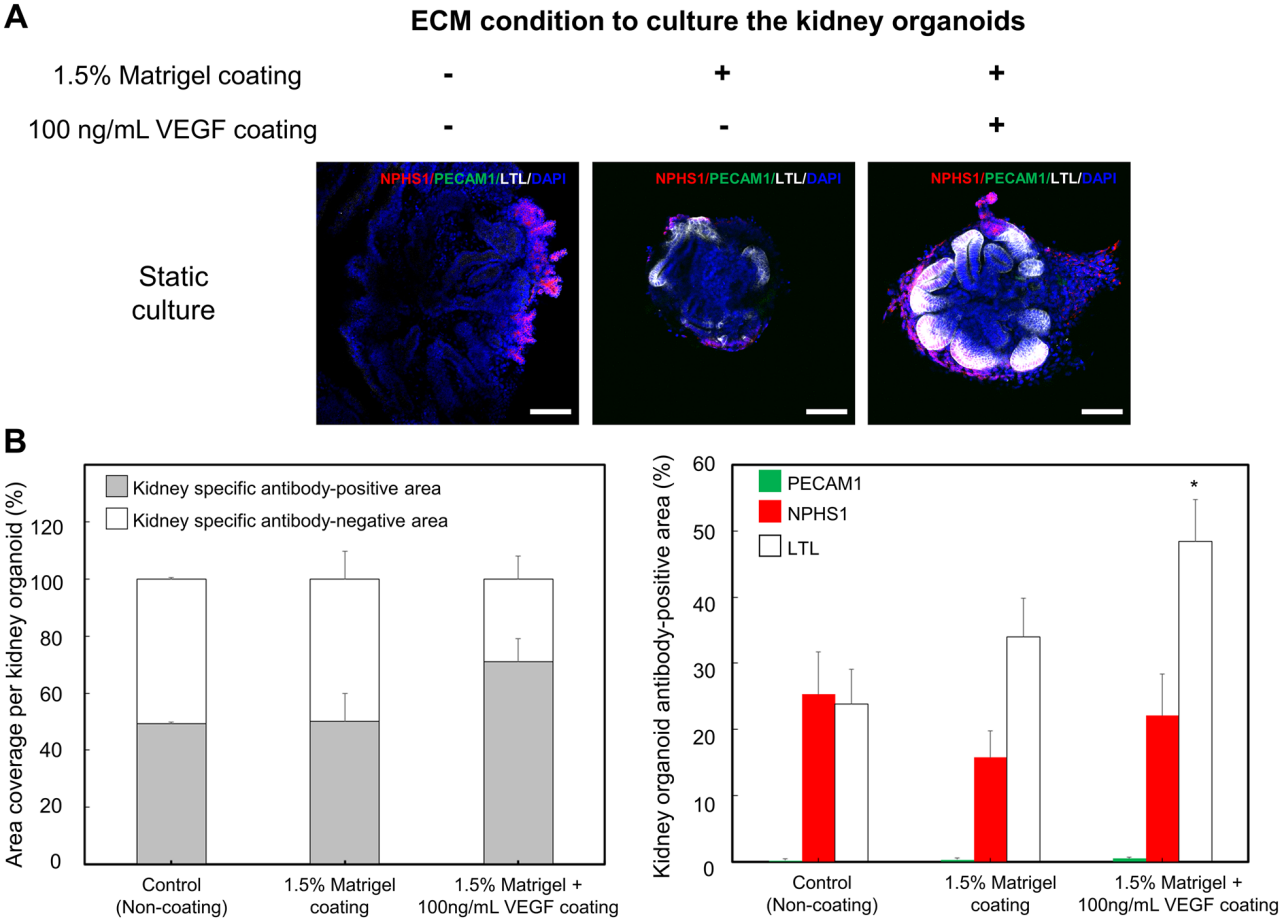
Effect of ECM on the differentiation of kidney organoids in a static culture condition. **A** Fluorescent images of immunostained kidney organoids cultured with various conditions of ECMs for 6 days. The cells were immunostained by NPHS1 (podocyte, red), PECAM1 (vascular endothelial cell, green), LTL (proximal tubules, white), and DAPI (nuclei, blue). **B** Quantitative analysis of kidney antibody-specific markers from kidney organoids (* indicates *p*<0.05 for experimental group vs. control group). Scale bars are 100 μm

To optimize ECM conditions for blood vessel formation of kidney organoids in a static condition, we coated 1.5% Matrigel and 1.5% Matrigel containing 100 ng/mL of VEGF on the substrate of a kidney organoid-on-a-chip. After cultured in the kidney organoid-on-a-chip for 6 days (differentiation day 22), specific markers of the kidney were examined (Fig. 3A). We analyzed the kidney antibody-specific area per organoids, showing that kidney antibody-specific area was the largest in a 1.5% Matrigel containing 100 ng/mL VEGF-coated substrate. The adherent 1.5% Matrigel containing with 100 ng/mL VEGF substrate led to enhanced peripheral expression of the vascular marker (PECAM1) and proximal tubule marker (LTL) within 1 week in a static condition, compared with expression in a non-adherent (e.g., glass substrate) and 1.5% Matrigel substrate. Furthermore, we observed that the expression of NPHS1, a marker of podocytes, was not significantly different in all groups, while LTL, a marker of proximal tubule, was higher expression in a 100 ng/mL VEGF-containing group. Additionally, an analysis of the PECAM1, a marker of the blood vessel formation, showed that PECAM1 was slightly expressed in an ECM-coated group than non-coating group (Fig. 3B). The expression levels of PECAM1-positive cells were 0.19 ± 0.15%, 0.34 ± 0.17%, and 0.53 ± 0.16%, respectively. Among three groups, the group coated with 1.5% Matrigel containing 100 ng/mL VEGF showed the highest expression as compared to other groups. The formation of the blood vessel is a process in which the embryo angioblasts differentiated from mesodermal cells are organized from a primitive network [28]. Although the molecular mechanism responsible for blood vessel formation has not been fully understood, a number of studies have indicated that a VEGF was generally regarded as a principal regulator in the differentiation of ECs and vascular development [29–32]. Furthermore, the previous studies have also demonstrated that a VEGF induced EC differentiation and vascularization of the tissues in in vivo and in vitro experiments [33–35]. In our study, we found the enhanced PECAM1-positive expression in a 1.5% Matrigel containing 100 ng/mL VEGF-coated substrate within 6 days. In addition, the previous reports have indicated that ECs played a significant role in blood vessel formation [36]. ECs are generally identified through the presence of specific markers, such as PECAM1, CD34, and VE-cadherin [37]. Our data showed that even in static condition, the treatment of VEGF improved the expression of PECAM1, suggesting that an ECM containing a VEGF could be a crucial factor for the differentiation of hPSCs into the ECs in a kidney organoid and formation of the blood vessel in vitro. Therefore, we confirmed that the differentiation system supplemented with multiple stimuli factors was more efficient for development and maturation of the kidney organoids.

### Differentiation and maturation of kidney organoids in a fluidic flow condition

**Fig. 4:**
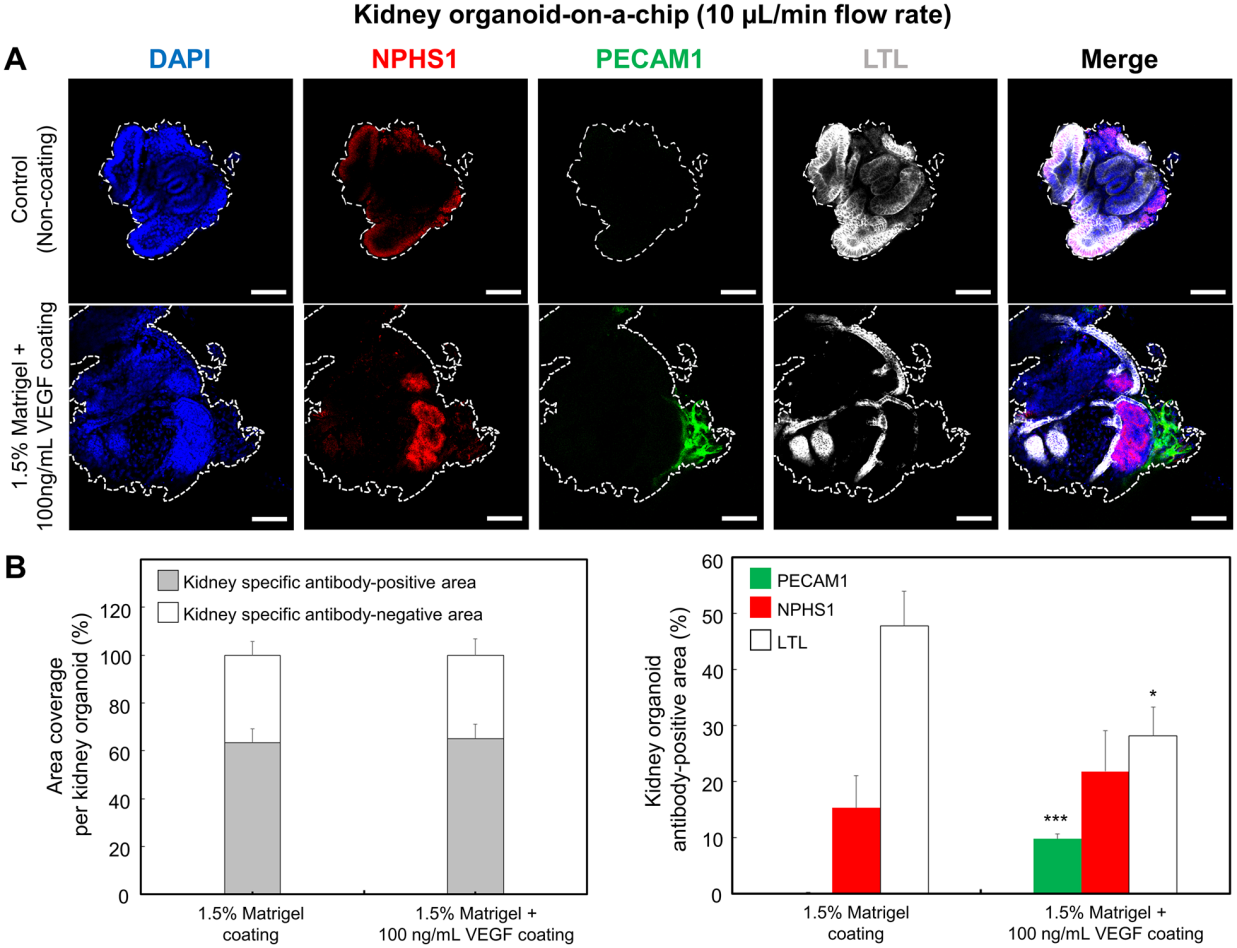
Effect of the shear stress on the differentiation of kidney organoids in a kidney organoid-on-a-chip. **A** Fluorescent images showing hPSCs-derived kidney organoid-related cell differentiation under a shear stress condition. Kidney organoids were cultured within kidney organoid-on-a-chip for 6 days. After culturing for 6 days, the cells were stained for NPHS1 (podocyte, red), PECAM1 (vascular endothelial cell, green), LTL (proximal tubules, white), and DAPI (nuclei, blue) to identify the kidney-related cells, respectively. **B** Quantitative analysis of the kidney-related markers from kidney organoids. (* indicates *p *< 0.05 for LTL expression of Matrigel-coated group vs. Matrigel/VEGF-coated group, *** indicates *p *< 0.001 for PECAM expression of Matrigel-coated group vs. Matrigel/VEGF-coated group). Scale bars are 100 μm

To determine the effect of the flow rate on maturation of the kidney organoids, we cultured kidney organoids on the adherent 1.5% Matrigel containing with 100 ng/mL VEGF in a kidney organoid-on-a-chip. The advanced RPMI 1640 medium supplemented with 1% l-glutamine and B-27 supplement were perfused at a rate of 10 µL/min (1.31 ⋅ 10^−5^ dyne/cm^3^ shear stress) in a kidney organoid-on-a-chip system for 6 days using the syringe pump. In a fluidic flow culture condition, the average of kidney-related area was not significantly difference regardless of ECM coating conditions. In contrast, we observed higher expression of NPHS1-positive and PECAM1-positive kidney organoids cultured under a flow culture condition. To quantify their blood vessel differentiation, we evaluated the confocal images of whole kidney organoids using the Image J software. We found that PECAM1-positive ECs increased in number to 8.82% of the cell population in a Matrigel/VEGF-coated kidney organoid-on-a-chip as compared to 0.18% in Matrigel-coated kidney organoid-on-a-chip (Fig. 4A, B). Furthermore, even in same ECM coating conditions, the kidney organoids in flow culture conditions (PECAM1-positive cells: 8.82%) were significantly (p < 0.01) increased vascular-related cells as compared to the kidney organoids in static culture conditions (PECAM1-positive cells: 0.53%). Thus, VEGF combined other biomechanical stimuli can synergistically ameliorate the formation of vascularization of the kidney organoids. In addition, compared with the Matrigel-coated chips, the foot-process-like structure seemed more prominent in a Matrigel/VEGF-coated kidney organoid-on-a-chip. These observations indicate that kidney organoids cultured under a fluidic flow culture condition show enhanced vascularization and foot process maturation. Taken together, the results demonstrated that the fluidic flow-induced shear stress promoted the differentiation of hPSCs into the vascular EC lineage. We confirm that the fluidic flow is a critical environment factor that facilitates the formation of blood vessel and maturation in kidney organoids in vitro. Under static conditions, the kidney organoids develop the more limited vasculature and tubular epithelia often have immature gene expression with analogous to that of early stage of kidney organoids. It has been known that the mechanical and physical forces were involved in organogenesis in an early development, as previously described [38]. In particular, the fluidic flow-induced shear stress is also known to play an important role in the development and differentiation of various types of the stem cells including embryonic stem cells and endothelial progenitor cells [38]. In our results have illustrated that the growth factors (e.g., VEGF) can induce hPSCs into an EC lineage. However, it shows that the effect of the biochemical stimuli alone on an endothelial differentiation has not enough to maturation of kidney organoids. This lack of the vascular network in hPSCs-induced kidney organoids might, in part, be due to the lack of microenvironment stimulation that could serve to promote the kidney differentiation in vivo. Blood vessels are constantly exposed to the shear stress. The previous studies have suggested that the laminar shear stress was responsible for the phenotype modulation, vascular remodeling, and EC differentiation [39–41]. In our study, we demonstrated a significantly increase in PECAM1-positive protein levels in hPSCs that could induce the kidney organoids exposed to the shear stress in a kidney organoid-on-a-chip. This result was consistent with the findings from other previous studies that examined the effect of the shear stress on the cell differentiation, as previously described [40, 42, 43]. These findings suggest that the kidney organoids subjected to the appropriate combination of adherent ECM, culture medium, and shear stress in vitro can form increasingly mature and perfusable vasculature.

### Analysis of drug-induced nephrotoxicity in a kidney organoid-on-a-chip

**Fig. 5:**
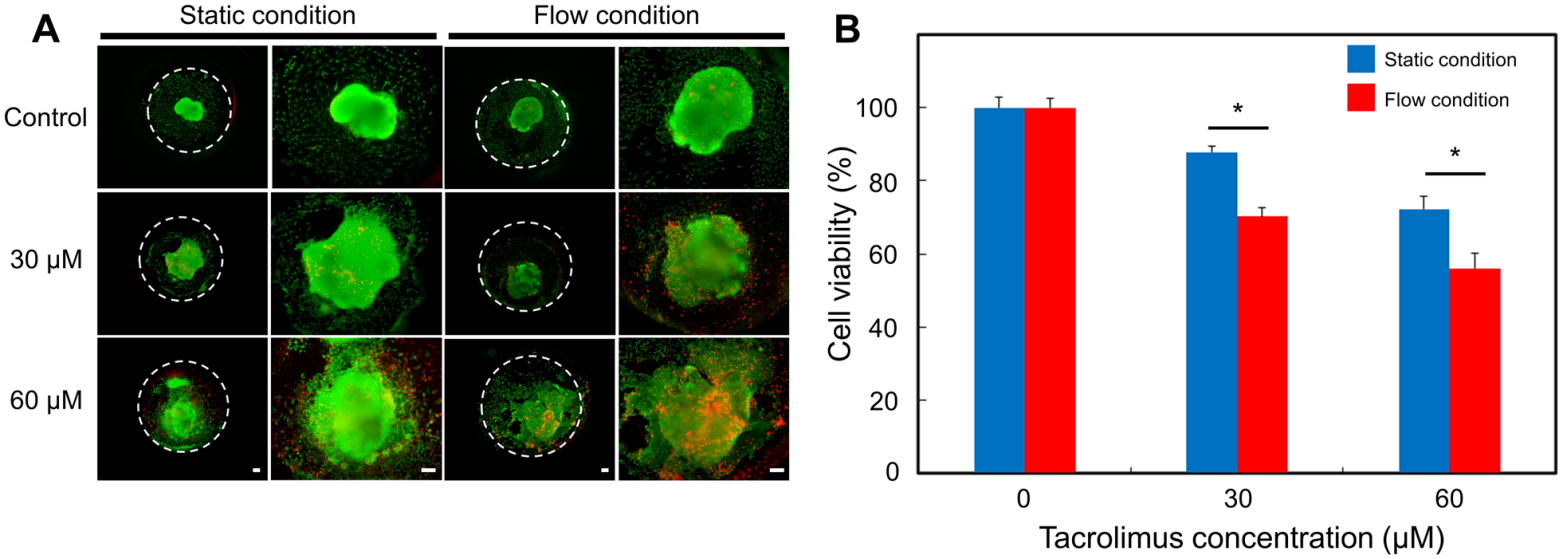
Cell viability of tacrolimus-treated hPSCs-derived kidney organoids. **A** Representative image of live/dead staining. Scale bar is 100 μm. **B** Viability of kidney organoids evaluated by CCK assay (n = 3, * indicates *p *< 0.05 for static condition vs. flow condition in each concentration of tacrolimus)

A major goal in developing a biomimetic organoid-on-a-chip is the development of an in vitro model that can more reliably predict drug toxicity in a human. We used a kidney organoid-on-a-chip system to investigate the response of the kidney organoids to different dosages of the nephrotoxicity drug. Since our kidney organoid-on-a-chip systems can preserve and maintain the mature formation of kidney organoids, it can serve as a useful in vitro model for predicting in vivo drug nephrotoxicity. We investigated the toxicity at different concentrations for nephrotoxic drug, known to tacrolimus, a calcineurin inhibitor, which was currently the main calcineurin inhibitor used clinically as an immunosuppressive agent in organ transplantation or glomerulonephritis [44, 45]. Despite the therapeutic benefits of tacrolimus, its use is limited due to its nephrotoxicity. The drug, tacrolimus, is selected as based on the criteria that (i) it is well-characterized nephrotoxic drug; (ii) it span over a range of toxic concentrations [46]. Organoids were perfusion- or static-cultured in the kidney-on-a-chip for 7 days prior to treatment at different concentrations of tacrolimus. We administered 30 and 60 µM concentrations of nephrotoxic drug, tacrolimus, *via* injection into the kidney organoid-on-a-chip system and static culture device. After 24 h, based on the nephrotoxicity profiles generated by the CCK-8 assay, we estimated the viability for each concentration. The viability of the kidney organoids in static culture conditions after 30 (88 ± 0.06%) and 60 µM (72 ± 0.06%) of tacrolimus treatments were significantly higher than those of the organoids in a fluidic culture condition on the corresponding concentrations (30 µM: 71 ± 0.06%, 60 µM: 51 ± 0.06%, Fig. 5B). In addition, a live/dead analysis revealed that the proportion of the dead cells was increased in a tacrolimus dose-dependent manner (Fig. 5A). Based on these results, tacrolimus increased cellular injury in both culture conditions in a dose dependent manner. In addition, kidney organoids cultured under fluidic conditions appeared higher drug sensitivity to various concentrations of tacrolimus than static condition cultured organoids. In general, in drug toxicity studies, the cellular maturity and microenvironment is crucial for prediction of initial toxic responses. Maturation of organoids are dependent on communications between cells, which includes paracrine and autocrine signals, as well as biomechanic and chemotactic processes [47]. These processes influence the proliferation, migration, and differentiation of the kidney cells. However, currently used in vitro models do not adequately predict in vivo observed effects, predominantly due to an insufficient differentiation of the organ’s cellular microenvironment [48]. A number of the studies demonstrated that the nephrotoxicity of drugs sensitively affected by maturity of organoids [49, 50]. In our results, the kidney organoids cultured in our fluidic flow conditions were sensitively responded to drug-mediated nephrotoxicity as compared to the static culture, suggesting that our kidney organoid-on-a-chip system could be a powerful tool for the nephrotoxicity analysis of the drugs.

## Conclusions

We developed a kidney organoid-on-a-chip to enable the control of the blood vessel differentiation of a kidney organoid. We investigated the effect of the mechanical properties on vascularization in vitro, confirming the critical role of mechanical stimuli in inducing the blood vessel. Moreover, our findings raise the possibility that the combinatorial effects of VEGF and shear stress can induce the higher expression of EC surface markers in hPSCs-derived kidney organoids. These findings suggest that the formation of the blood vessel from the kidney organoids can be regulated not only by biochemical stimuli (e.g., VEGF), but also by biomechanical stimuli (e.g., shear stress). This study may serve as a starting point in understanding the combinatorial effects of the biochemical and biomechanical factor on the vascularization of the kidney organoids in a kidney organoid-on-a-chip system. Further studies will undoubtedly provide more insight into formation of the blood vessel *via* the biochemical and biomechanical combinatorial stimuli, which will have implications for various research areas regarding kidney organoid-mediated neovascularization.

## Data Availability

The authors have no data to share since all data are shown in the submitted manuscript.

## Abbreviations

hPSCs: Human pluripotent stem cells
ECM: Extracellular matrix
ECs: Endothelial cells
VEGF: Vascular endothelial growth factor
